# A SQUAMOSA promoter binding protein‐like transcription factor controls crop ideotype for high productivity in barley

**DOI:** 10.1002/pld3.450

**Published:** 2022-09-09

**Authors:** Shengming Yang, Megan Overlander‐Chen, Craig H. Carlson, Jason D. Fiedler

**Affiliations:** ^1^ USDA‐ARS Cereals Research Unit Edward T. Schafer Agricultural Research Center Fargo ND USA; ^2^ Department of Plant Sciences North Dakota State University Fargo ND USA; ^3^ Department of Plant Pathology North Dakota State University Fargo ND USA

In cereal crops, erect leaf (small leaf angle) is a desirable trait that allows for dense planting through optimization of light capture, thus increasing photosynthesis efficiency and grain yield with a higher leaf area index. Therefore, understanding the genetic regulation of leaf angles is crucial in the development of high‐yielding crop cultivars. In the present study, we cloned the *Liguleless 1* (*Lig1*) gene in barley (*Hordeum vulgare* subsp. *vulgare*), which encodes SQUAMOSA promoter binding protein‐like 8 (SPL8), elucidating a conserved mechanism underlying regulation of leaf angle in cereals.

Barley is the fourth most important cereal crop and a valuable model monocot for genetic research. A spontaneous mutant in an unknown cultivar, *liguleless1* (*lig1*), is deficient in the formation of the ligule and auricle, exhibiting smaller leaf angles and a compact architecture (Figure 1a–c). An introduced mutant, BW483, was made by introgression of the *lig1* mutation into the two‐rowed *cv*. Bowman (Druka et al., 2011). On the single‐plant basis, the near‐isogenic pair of Bowman and BW483 showed similar yield and agronomic traits, with a slightly later heading in BW483 (Figures 1d and S1) (Druka et al., 2011). An erect leaf phenotype is generally coupled with undesirable agronomic traits, such as reduced grain size, fertility, tiller number, and tolerance to stress (Zhang et al., 2021), and plants with erect leaf phenotypes are only useful if elite yield components are well maintained. Therefore, *lig1* provides a valuable resource for yield improvement with dense planting in barley.

**FIGURE 1:**
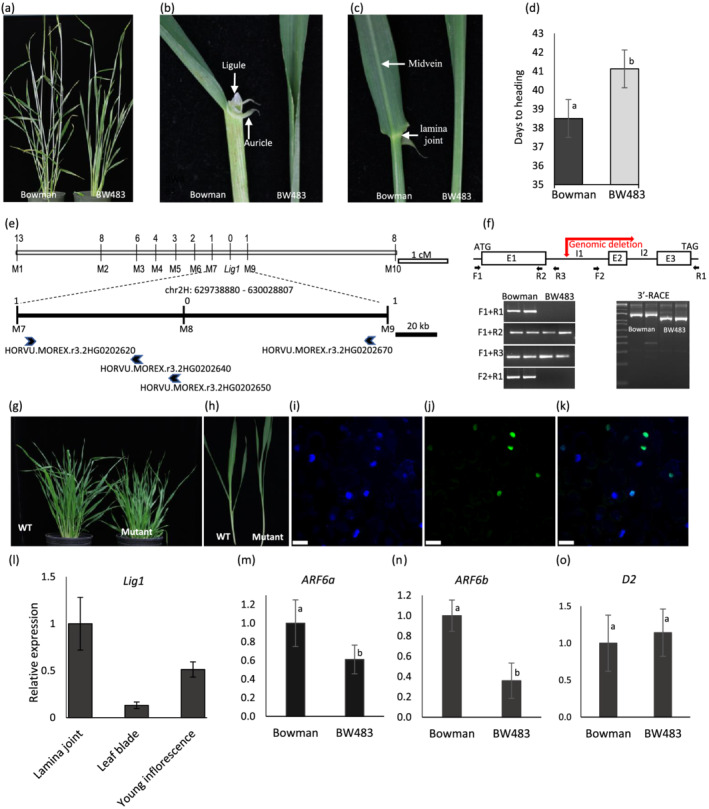
Map‐based cloning and functional characterization of *Lig1*. A compact architecture caused by erect leaves was observed in BW483 (a). The auricle, ligule, laminal joint, and leaf midvein are missing in the mutant (b, c). Scale bars in (a)–(c) are 15, 1, and 1 cm, respectively. Heading date was slightly delayed in BW483 (d). *Lig1* is located on 2H, delimited to an ~0.8 cm region (e). Numbers above the linkage group indicate recombination breakpoints. Four protein‐coding genes were identified in the *Lig1* region spanning ~290 kb (e). The *HvSPL8* coding region contains three exons (rectangles) and two introns (straight lines) (f). Various primers indicated by arrows were used to analyze HvSPL8 alleles with two plants each for Bowman and BW483 (f). Sequencing 3′‐RACE products revealed a large genomic deletion in the mutant, denoted by a red right‐angle arrow (f). Functional validation of *HvSPL8* was conducted using CRISRP‐mediated mutagenesis. Targeted knockout of *HvSPL8* phenocopied the *lig1* mutant for the whole plant (g) and a single tiller (h). Localization of Lig1 to the nucleus apparent in overlay of DAPI and GFP images (i–k). *Lig1* is highly expressed in the lamina joint (l). *HvARF6* genes were downregulated (m, n), but HvD2 expression was unaffected in the mutant (o). Different letters on bar graphs indicate significance at 0.01 level by *t* test.

To identify the *Lig1* gene, we conducted map‐based cloning using 257 F_2_ plants derived from the cross between Bowman and BW483. Of the tested F_2_ individuals, 196 showed normal plant architecture, and 61 were liguleless. The 3:1 ratio (*χ* ^2^ = 0.219, *df* = 1, and *p* = 0.64) suggested that the *lig1* mutation is monofactorial recessive. For immediate gene localization and marker discovery, 46 F_2_ plants (23 each for wild type and ligueleless) were genotyped with the barley 50k iSelect SNP Array. Consistent with the previous result (Rossini et al., 2006), the *Lig1* gene was anchored to chromosome 2H, and linked SNPs were converted to semi‐thermal asymmetric reverse PCR (STARP) markers for fine mapping (Table S1). Cosegregating with the SNP maker M8, the *Lig1* gene was delimited within an ~290 kb region by M7 and M9 (Figure 1e). Annotation indicated that four protein‐coding genes reside within the *Lig1* region (Table S2). Of those, *HORVU.MOREX.r3.2HG0202650* encodes a putative SQUAMOSA promoter binding protein‐like (SPL) transcription factor sharing high similarity to AtSPL8, ZmLg1, OsLG1, and TaSPL8 (Figure S2) (Lee et al., 2007; Liu et al., 2019; Moreno et al., 1997; Unte et al., 2003). Therefore, we named *HORVU.MOREX.r3.2HG0202650 HvSPL8*.

SPL genes encode plant‐specific transcription factors and play vital regulatory roles in various developmental processes, such as the transition from the vegetative to reproductive phase mediated by miR156. As a non‐miR156 target, AtSPL8 is a tissue‐dependent regulator affecting reproductive development, trichome formation on sepals, and stem filament elongation (Unte et al., 2003). In wheat, TaSPL8 is involved in ligule and auricle development (Liu et al., 2019). OsLg1 also plays a critical role in shaping of a closed panicle and seed shattering during rice domestication, in addition to regulating lamina joint formation (Ishii et al., 2013). Therefore, SPL8 may distinctly function between monocots and dicots. The *HvSPL8* gene contains three exons and two introns (Figure 1f). In BW483, we amplified only the first exon and part of intron 1, while the last two exons and the full‐length coding region of *Hvspl8* were not present (Figure 1f). To capture the mutation in *Hvspl8*, we conducted 3′‐rapid amplification of cDNA ends (RACE) (Figure 1f). Sequencing of RACE products indicated that a ⁓10 kb genomic deletion (chr5H: 629747100‐629757046) occurred at the first intron, and the last two exons were deleted in BW483 (Figure 1f). However, the neighboring gene, *HORVU.MOREX.r3.2HG0202640* encoding a putative universal stress family protein, remains intact in the mutant (Figure S3). Therefore, the *HvSPL8* gene was selected as the candidate of *Lig1*.

Using the barley genotype Golden Promise, we targeted the first exon of *HvSPL8* for CRISPR‐mediated mutagenesis with the *Agrobacterium tumefaciens* strain AGL1 and the JD633 vector (Debernardi et al., 2020; Harwood, 2014). All recovered T_0_ or M_0_ plants (*n* = 6) were defective in ligule and auricle formation and displayed a compact architecture (Figure 1g,h). DNA sequencing revealed five different mutant alleles, and at least one allele was discovered in each transgenic plant (Figure S4). All mutant alleles disrupted the HvSPL8 function completely. The liguleless phenotype was also confirmed in the T_1_ or M1 generation (*n* > 90 plants/transgenic line). The slightly delayed heading of the M1 plants validated that HvSPL8 facilitates reproductive phase transition, although it is not a target for miR156 (Figure S5). Therefore, we concluded that *HvSPL8* is indeed the *Lig1* gene.

To determine its subcellular localization, we fused HvSPL8 with a green fluorescent protein (GFP) under the CaMV 35S promoter using the pSite‐2NB vector. Transient expression of HvSPL8‐GFP in *Nicotiana benthamiana* revealed that the fusion protein was co‐localized with 4′,6‐diamidino‐2‐phenylindole (DAPI)‐stained nuclei (Figure 1i–k), corroborating its role as a transcription factor. The BaRTv1.0 transcript dataset has been constructed to spatiotemporally quantify gene expression in different barley tissues (Rapazote‐Flores et al., 2019). Overall, low levels of *HvSPL8* expression were shown by BaRTv1.0 across all the tissues, but relatively higher expression levels were detected in inflorescence tissues, consistent with the involvement of *HvSPL8* in reproductive development (Figure S6 and Table S3). Quantitative reverse transcription PCR (qRT‐PCR) using 4‐week‐old seedlings showed that *HvSPL8* is highly expressed in the lamina joint where the ligule and auricle form (Figure 1l). The fine‐tuned local expression of *HvSPL8* is likely the key to the compact architecture without causing other deleterious traits in BW483.

Genetic and functional studies revealed that various genes regulate leaf angle through phytohormone signaling pathways. In rice, disrupted biosynthesis of brassinosteroid (BR) results in a reduced leaf angle, and auxin is also involved in the regulation of leaf angle by modulating secondary cell wall biosynthesis in lamina joint tissues (Hong et al., 2005; Huang et al., 2021). OsARF6 is a critical player in auxin signaling, while OsD2 is a cytochrome P450 whose catalytic activity is required for BR biosynthesis. To determine if the SPL‐mediated development of the ligule and auricle involves BR or auxin signaling, we analyzed the expression of *HvARF6s* and *HvD2* (Table S4). Two potential orthologs of *AtARF6* were identified in barley, and both of them were downregulated at the lamina joint in BW483 (Figure 1m,n). However, expression of *HvD2* was not affected by the *lig1* mutation (Figure 1o). Therefore, HvSPL8 may regulate leaf angle through controlling of the auxin signaling pathway, which is indispensable for secondary cell wall biosynthesis.

In summary, we cloned and functionally validated the *Lig1* gene regulating leaf angle in barley. It was indicated that *Lig1* encodes a plant‐specific transcription factor, HvSPL8. Cloning of *Lig1* provide a target for gene manipulation to increase spike numbers especially under dense planting conditions. Small leaf angle due to the loss of Lig1 function leads to efficient light interception, and erect leaves also allow increased light shedding to lower leaves, thereby improving canopy photosynthesis, which in turn facilitates crop productivity.

## CONFLICTS OF INTEREST

The authors declare no conflicts of interest.

## AUTHOR CONTRIBUTIONS

S.Y. designed the experiments and wrote the first draft of the manuscript. S.Y. and M.O.C. developed the populations. M.O.C. conducted genetic mapping, gene validation, and gene function characterization. C.H.C. and J.D.F. contributed new reagents/analytic tools. S.Y. and M.C.O. analyzed the data. All authors commented on previous versions of the manuscript, and read and approved the final manuscript. The author responsible for distribution of materials integral to the findings presented in this article in accordance with the policy described in the Instructions for Authors (https://academic.oup.com/plphys) is S.Y. (shengming.yang@usda.gov).

## Supporting information


**Figure S1.** Comparison of agronomic traits between WT Bowman and its NIL BW387 carrying the *lig1* mutation. No significant difference was identified in height (A, n = 10), tiller number/plant (B, n = 20), seed/number/spike (C, n = 20), and 100‐seed weight (D, n = 7). Same letters on bar graphs indicate insignificant at 0.05 level by T‐test.
**Figure S2.** Sequence alignment of HvSPL8 (HORVU.MOREX.r3.2HG0202650), TaSPL8 (TraesCS2D01G502900), OsSPL8 (Os04 g56170), ZmLG1 (U89496), and AtSPL8 (At1g02065). Protein sequences (A) and the SQUAMOSA promoter binding protein (SBP) domains sequences (B) were aligned and depicted using ClustalX. The two zinc‐finger structures (Zn1 and Zn2) and nuclear localization signal (NLS) are denoted.
**Figure S3.** Genomic deletion in the BW483 mutant. An ⁓10 kb deletion (denoted by two read arrows and a red line) in *HORVU.MOREX.r3.2HG0202650* (*HvSPL8*) was identified by 3′‐RACE in BW483 mutant, which is ⁓7.5 kb apart from the putative 5′UTR of *HORVU.MOREX.r3.2HG0202640*. The deletion eliminates the 2nd and 3rd coding exons, disrupting the HvSPL8 function totally in BW483. Primers used for amplification of *HvSPL8* sequences were indicated by black arrows. Gene models on chromosome were denoted by blue arrows combing with small rectangles (coding exon) and blue lines (introns and UTRs). Blue arrows represent the transcriptional directions. E, coding exon; I, intron. UTR, untranslated region.
**Figure S4.** Gene mutations highlighted by red colons (deletion) and letters (insertion or substitution). All mutations lead to an early stop codon, which disrupts the Lig1 function. The PAM sequence was highlighted in yellow.
**Figure S5.** Delayed heading in the *Lig1*‐knockout mutant. On the average, homozygous mutants in M1 (n = 15) is 5.3 days later in heading than WT Golden Promise (n = 15). Different letters on bar graphs indicate significant at 0.05 level by T‐test.
**Figure S6.** Spaciotemporal analysis of the *Lig1* gene using the BaRTv1.0 data. TPM, transcripts per million.

**Table S1.** Marker sequences used for genetic mapping and mutation analysis of the *Lig1* gene.
**Table S2.** Predicted genes in the *Lig1* region.
**Table S3.** Tissue sampling for Barley Reference Transcript (BaRTv1.0) Dataset (Jayakodi et al., 2020).
**Table S4.** Primer sequences used for qRT‐PCR analysis.
