# Supplementary material for: A SQUAMOSA promoter binding protein‐like transcription factor controls crop ideotype for high productivity in barley
Source: Plant Direct. 2022 Sep 9;6(9):e450. doi: 10.1002/pld3.450 (PMC9477381; doi:10.1002/pld3.450)
Supplement: Supplementary file 1 — Figure S1. Comparison of agronomic traits between WT Bowman and its NIL BW387 carrying the lig1 mutation. No significant difference was identified in height (A, n = 10), tiller number/plant (B, n = 20), seed/number/spike (C, n = 20), and 100‐seed weight (D, n = 7). Same letters on bar graphs indicate insignificant at 0.05 level by T‐test. Figure S2. Sequence alignment of HvSPL8 (HORVU.MOREX.r3.2HG0202650), TaSPL8 (TraesCS2D01G502900), OsSPL8 (Os04 g56170), ZmLG1 (U89496), and AtSPL8 (At1g02065). Protein sequences (A) and the SQUAMOSA promoter binding protein (SBP) domains sequences (B) were aligned and depicted using ClustalX. The two zinc‐finger structures (Zn1 and Zn2) and nuclear localization signal (NLS) are denoted. Figure S3. Genomic deletion in the BW483 mutant. An ⁓10 kb deletion (denoted by two read arrows and a red line) in HORVU.MOREX.r3.2HG0202650 (HvSPL8) was identified by 3′‐RACE in BW483 mutant, which is ⁓7.5 kb apart from the putative 5′UTR of HORVU.MOREX.r3.2HG0202640. The deletion eliminates the 2nd and 3rd coding exons, disrupting the HvSPL8 function totally in BW483. Primers used for amplification of HvSPL8 sequences were indicated by black arrows. Gene models on chromosome were denoted by blue arrows combing with small rectangles (coding exon) and blue lines (introns and UTRs). Blue arrows represent the transcriptional directions. E, coding exon; I, intron. UTR, untranslated region. Figure S4. Gene mutations highlighted by red colons (deletion) and letters (insertion or substitution). All mutations lead to an early stop codon, which disrupts the Lig1 function. The PAM sequence was highlighted in yellow. Figure S5. Delayed heading in the Lig1‐knockout mutant. On the average, homozygous mutants in M1 (n = 15) is 5.3 days later in heading than WT Golden Promise (n = 15). Different letters on bar graphs indicate significant at 0.05 level by T‐test. Figure S6. Spaciotemporal analysis of the Lig1 gene using the BaRTv1.0 data. TPM, transcripts per mill [file PLD3-6-e450-s001.pptx]

## Slide 1
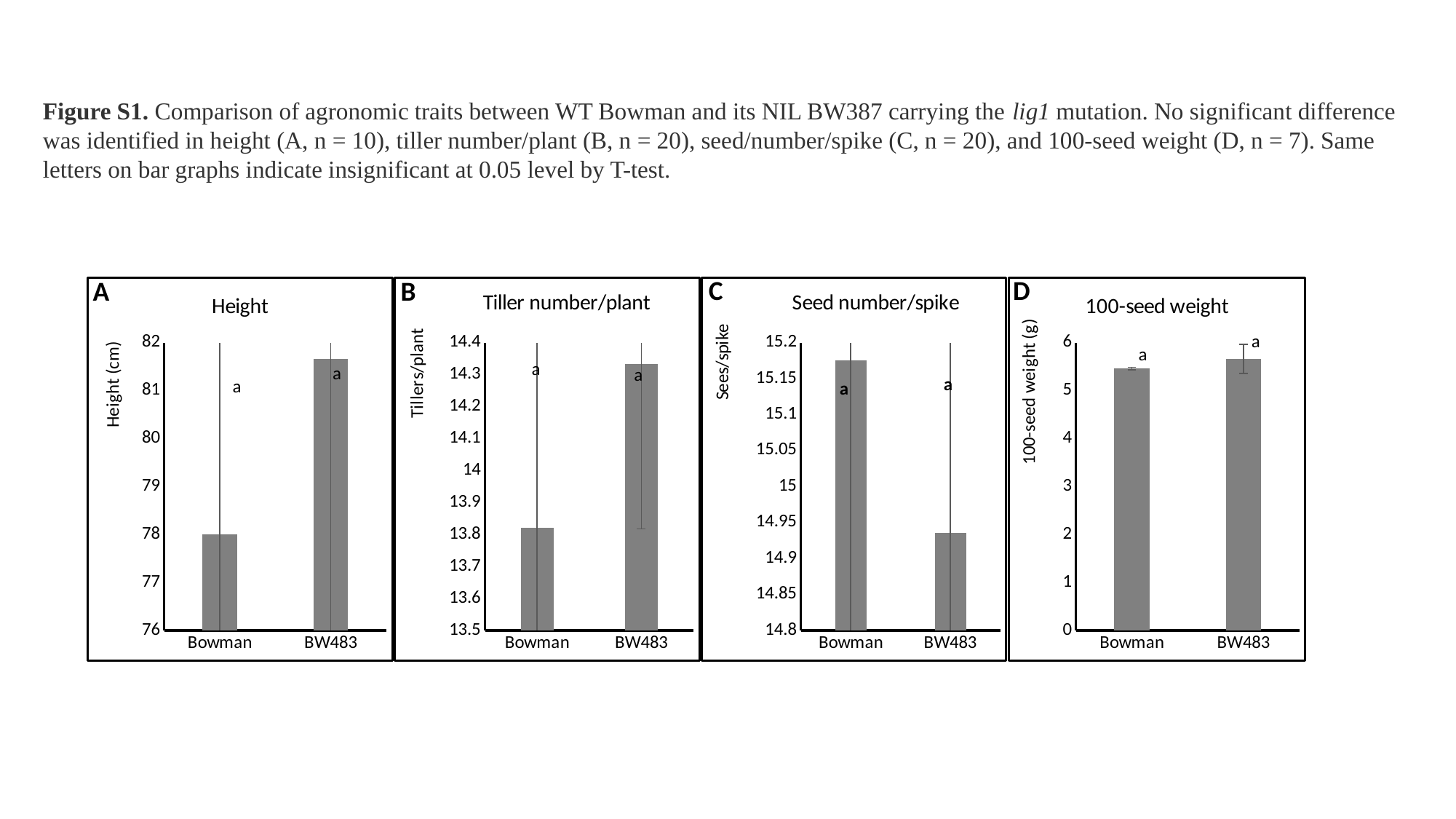

Figure S1. Comparison of agronomic traits between WT Bowman and its NIL BW387 carrying the lig1 mutation. No significant difference was identified in height (A, n = 10), tiller number/plant (B, n = 20), seed/number/spike (C, n = 20), and 100-seed weight (D, n = 7). Same letters on bar graphs indicate insignificant at 0.05 level by T-test.
C
D
A
B
### Chart: Height
| Category | Height (cm) |
|---|---|
| Bowman | 78.0 |
| BW483 | 81.66666666666667 |
### Chart: Tiller number/plant
| Category | # of tillers |
|---|---|
| Bowman | 13.82 |
| BW483 | 14.333333333333334 |
### Chart: Seed number/spike
| Category | Seeds/spike |
|---|---|
| Bowman | 15.175 |
| BW483 | 14.9354838709677 |
### Chart: 100-seed weight
| Category | |
|---|---|
| Bowman | 5.456666666666666 |
| BW483 | 5.66 |a
a
a
a
a
a
a
a

## Slide 2
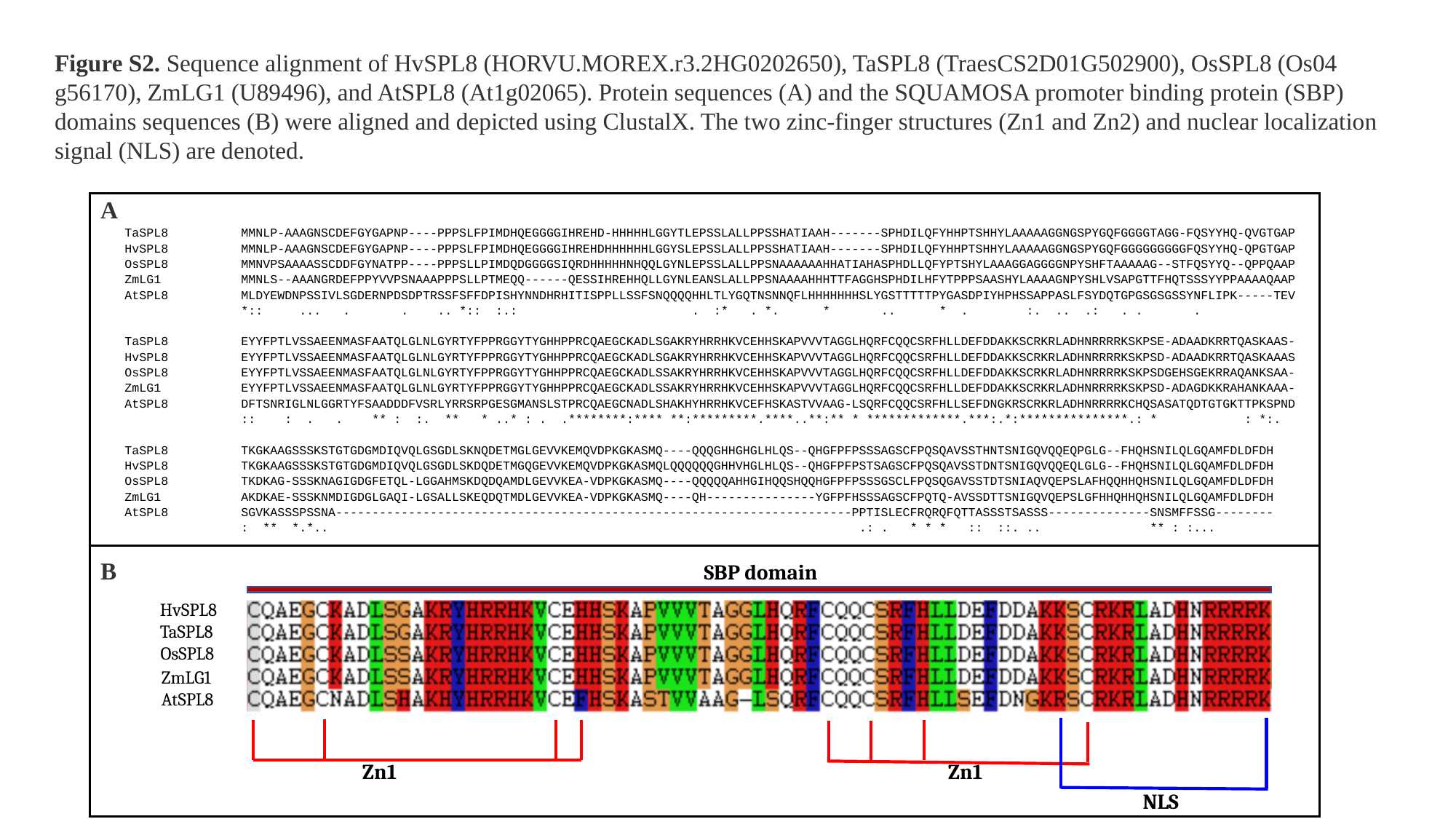

Figure S2. Sequence alignment of HvSPL8 (HORVU.MOREX.r3.2HG0202650), TaSPL8 (TraesCS2D01G502900), OsSPL8 (Os04 g56170), ZmLG1 (U89496), and AtSPL8 (At1g02065). Protein sequences (A) and the SQUAMOSA promoter binding protein (SBP) domains sequences (B) were aligned and depicted using ClustalX. The two zinc-finger structures (Zn1 and Zn2) and nuclear localization signal (NLS) are denoted.
A
TaSPL8 MMNLP-AAAGNSCDEFGYGAPNP----PPPSLFPIMDHQEGGGGIHREHD-HHHHHLGGYTLEPSSLALLPPSSHATIAAH-------SPHDILQFYHHPTSHHYLAAAAAGGNGSPYGQFGGGGTAGG-FQSYYHQ-QVGTGAP
HvSPL8 MMNLP-AAAGNSCDEFGYGAPNP----PPPSLFPIMDHQEGGGGIHREHDHHHHHHLGGYSLEPSSLALLPPSSHATIAAH-------SPHDILQFYHHPTSHHYLAAAAAGGNGSPYGQFGGGGGGGGGFQSYYHQ-QPGTGAP
OsSPL8 MMNVPSAAAASSCDDFGYNATPP----PPPSLLPIMDQDGGGGSIQRDHHHHHNHQQLGYNLEPSSLALLPPSNAAAAAAHHATIAHASPHDLLQFYPTSHYLAAAGGAGGGGNPYSHFTAAAAAG--STFQSYYQ--QPPQAAP
ZmLG1 MMNLS--AAANGRDEFPPYVVPSNAAAPPPSLLPTMEQQ------QESSIHREHHQLLGYNLEANSLALLPPSNAAAAHHHTTFAGGHSPHDILHFYTPPPSAASHYLAAAAGNPYSHLVSAPGTTFHQTSSSYYPPAAAAQAAP
AtSPL8 MLDYEWDNPSSIVLSGDERNPDSDPTRSSFSFFDPISHYNNDHRHITISPPLLSSFSNQQQQHHLTLYGQTNSNNQFLHHHHHHHSLYGSTTTTTPYGASDPIYHPHSSAPPASLFSYDQTGPGSGSGSSYNFLIPK-----TEV
 *:: ... . . .. *:: :.: . :* . *. * .. * . :. .. .: . . .
TaSPL8 EYYFPTLVSSAEENMASFAATQLGLNLGYRTYFPPRGGYTYGHHPPRCQAEGCKADLSGAKRYHRRHKVCEHHSKAPVVVTAGGLHQRFCQQCSRFHLLDEFDDAKKSCRKRLADHNRRRRKSKPSE-ADAADKRRTQASKAAS-
HvSPL8 EYYFPTLVSSAEENMASFAATQLGLNLGYRTYFPPRGGYTYGHHPPRCQAEGCKADLSGAKRYHRRHKVCEHHSKAPVVVTAGGLHQRFCQQCSRFHLLDEFDDAKKSCRKRLADHNRRRRKSKPSD-ADAADKRRTQASKAAAS
OsSPL8 EYYFPTLVSSAEENMASFAATQLGLNLGYRTYFPPRGGYTYGHHPPRCQAEGCKADLSSAKRYHRRHKVCEHHSKAPVVVTAGGLHQRFCQQCSRFHLLDEFDDAKKSCRKRLADHNRRRRKSKPSDGEHSGEKRRAQANKSAA-
ZmLG1 EYYFPTLVSSAEENMASFAATQLGLNLGYRTYFPPRGGYTYGHHPPRCQAEGCKADLSSAKRYHRRHKVCEHHSKAPVVVTAGGLHQRFCQQCSRFHLLDEFDDAKKSCRKRLADHNRRRRKSKPSD-ADAGDKKRAHANKAAA-
AtSPL8 DFTSNRIGLNLGGRTYFSAADDDFVSRLYRRSRPGESGMANSLSTPRCQAEGCNADLSHAKHYHRRHKVCEFHSKASTVVAAG-LSQRFCQQCSRFHLLSEFDNGKRSCRKRLADHNRRRRKCHQSASATQDTGTGKTTPKSPND
 :: : . . ** : :. ** * ..* : . .********:**** **:*********.****..**:** * *************.***:.*:***************.: * : *:.
TaSPL8 TKGKAAGSSSKSTGTGDGMDIQVQLGSGDLSKNQDETMGLGEVVKEMQVDPKGKASMQ----QQQGHHGHGLHLQS--QHGFPFPSSSAGSCFPQSQAVSSTHNTSNIGQVQQEQPGLG--FHQHSNILQLGQAMFDLDFDH
HvSPL8 TKGKAAGSSSKSTGTGDGMDIQVQLGSGDLSKDQDETMGQGEVVKEMQVDPKGKASMQLQQQQQQGHHVHGLHLQS--QHGFPFPSTSAGSCFPQSQAVSSTDNTSNIGQVQQEQLGLG--FHQHSNILQLGQAMFDLDFDH
OsSPL8 TKDKAG-SSSKNAGIGDGFETQL-LGGAHMSKDQDQAMDLGEVVKEA-VDPKGKASMQ----QQQQQAHHGIHQQSHQQHGFPFPSSSGSCLFPQSQGAVSSTDTSNIAQVQEPSLAFHQQHHQHSNILQLGQAMFDLDFDH
ZmLG1 AKDKAE-SSSKNMDIGDGLGAQI-LGSALLSKEQDQTMDLGEVVKEA-VDPKGKASMQ----QH---------------YGFPFHSSSAGSCFPQTQ-AVSSDTTSNIGQVQEPSLGFHHQHHQHSNILQLGQAMFDLDFDH
AtSPL8 SGVKASSSPSSNA-----------------------------------------------------------------------PPTISLECFRQRQFQTTASSSTSASSS--------------SNSMFFSSG--------
 : ** *.*.. .: . * * * :: ::. .. ** : :...
B
SBP domain
HvSPL8
TaSPL8
OsSPL8
ZmLG1
AtSPL8
Zn1
Zn1
NLS

## Slide 3
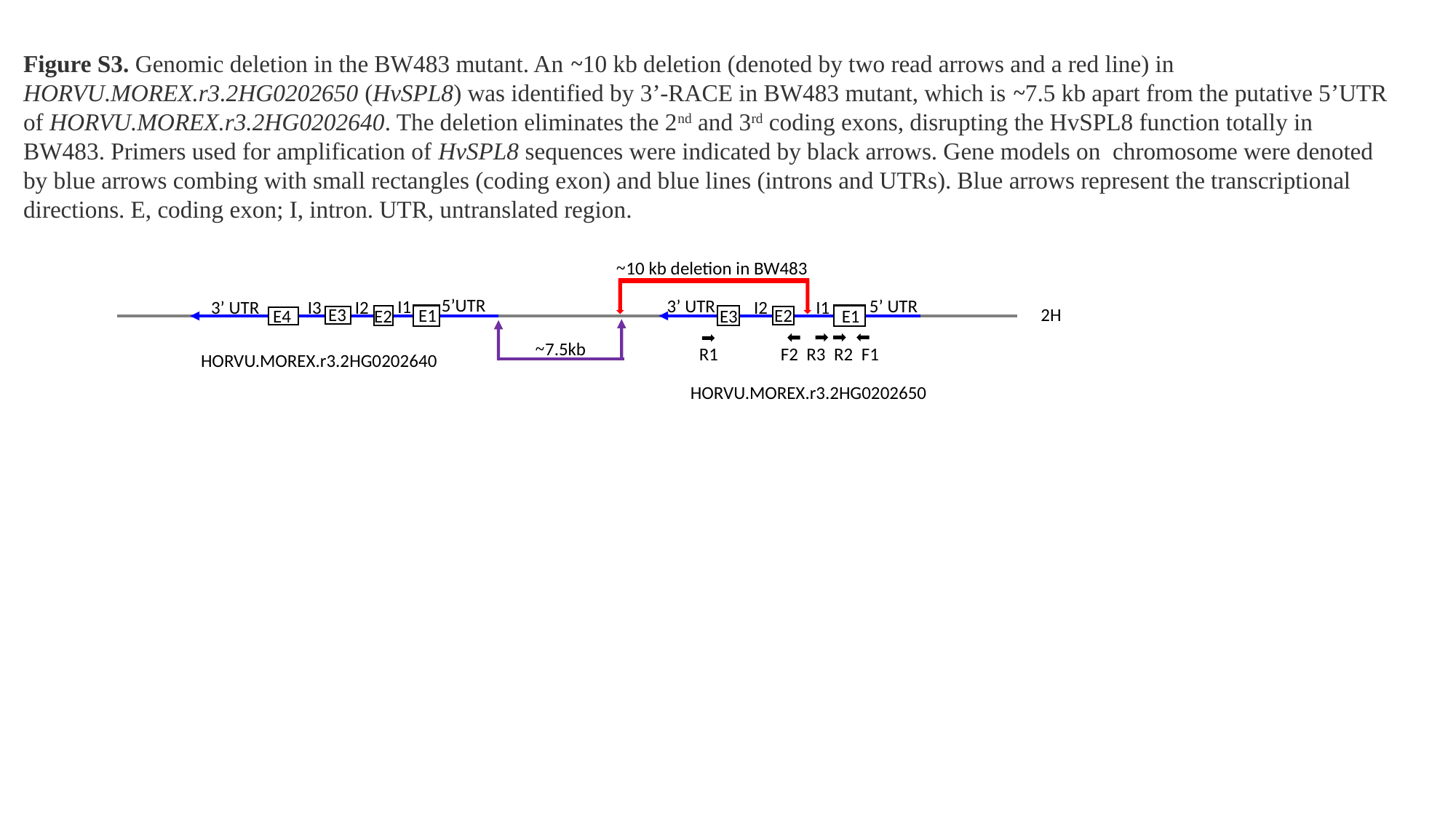

Figure S3. Genomic deletion in the BW483 mutant. An ⁓10 kb deletion (denoted by two read arrows and a red line) in HORVU.MOREX.r3.2HG0202650 (HvSPL8) was identified by 3’-RACE in BW483 mutant, which is ⁓7.5 kb apart from the putative 5’UTR of HORVU.MOREX.r3.2HG0202640. The deletion eliminates the 2nd and 3rd coding exons, disrupting the HvSPL8 function totally in BW483. Primers used for amplification of HvSPL8 sequences were indicated by black arrows. Gene models on chromosome were denoted by blue arrows combing with small rectangles (coding exon) and blue lines (introns and UTRs). Blue arrows represent the transcriptional directions. E, coding exon; I, intron. UTR, untranslated region.
⁓10 kb deletion in BW483
5’UTR
5’ UTR
3’ UTR
I1
I3
I2
3’ UTR
I2
I1
2H
E3
E1
E2
E2
E1
E4
E3
⁓7.5kb
R1 F2 R3 R2 F1
HORVU.MOREX.r3.2HG0202640
HORVU.MOREX.r3.2HG0202650

## Slide 4
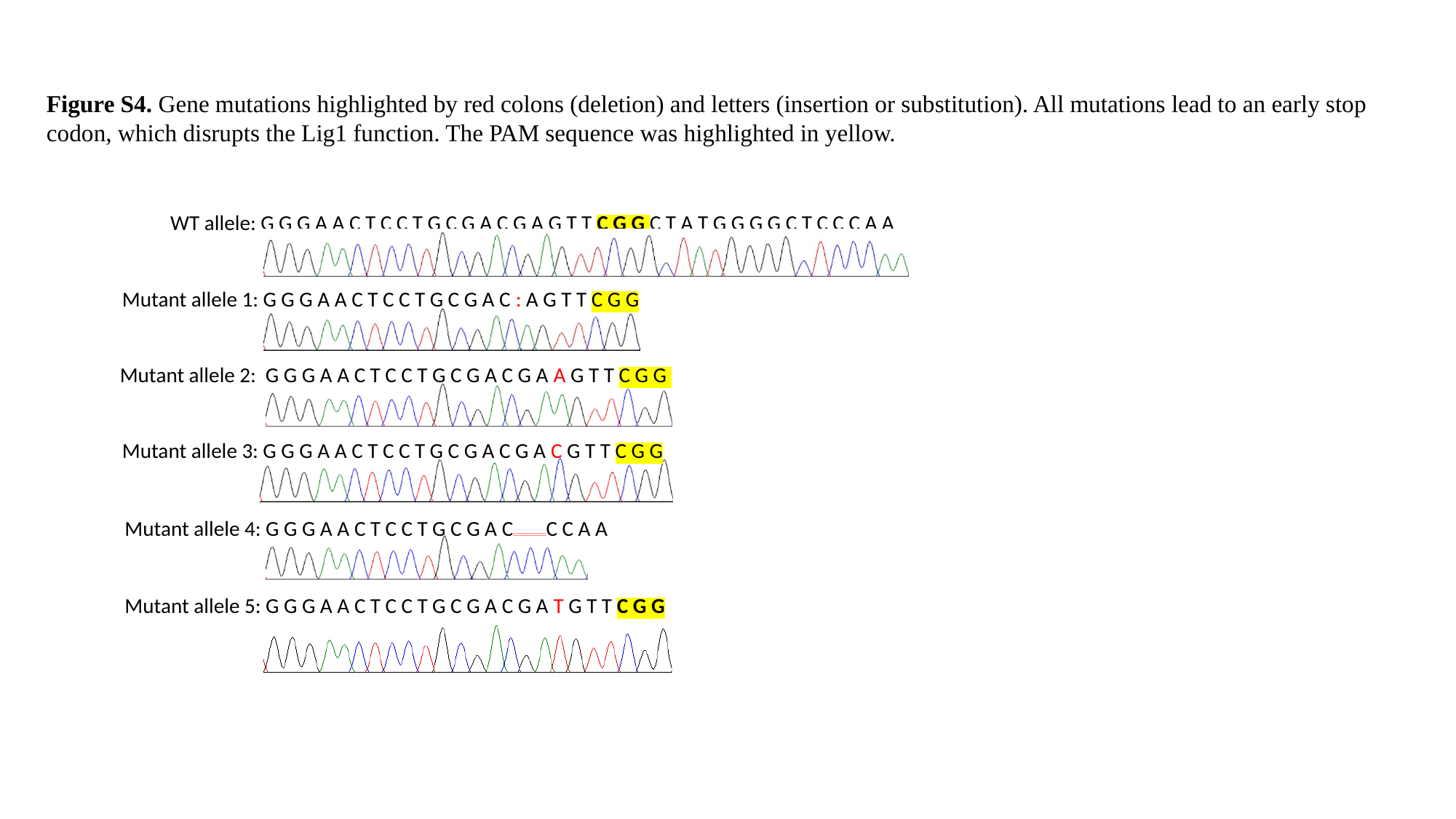

Figure S4. Gene mutations highlighted by red colons (deletion) and letters (insertion or substitution). All mutations lead to an early stop codon, which disrupts the Lig1 function. The PAM sequence was highlighted in yellow.
WT allele: G G G A A C T C C T G C G A C G A G T T C G G C T A T G G G G C T C C C A A
Mutant allele 1: G G G A A C T C C T G C G A C : A G T T C G G
Mutant allele 2: G G G A A C T C C T G C G A C G A A G T T C G G
Mutant allele 3: G G G A A C T C C T G C G A C G A C G T T C G G
Mutant allele 4: G G G A A C T C C T G C G A C::::::::::::::::::::C C A A
Mutant allele 5: G G G A A C T C C T G C G A C G A T G T T C G G

## Slide 5
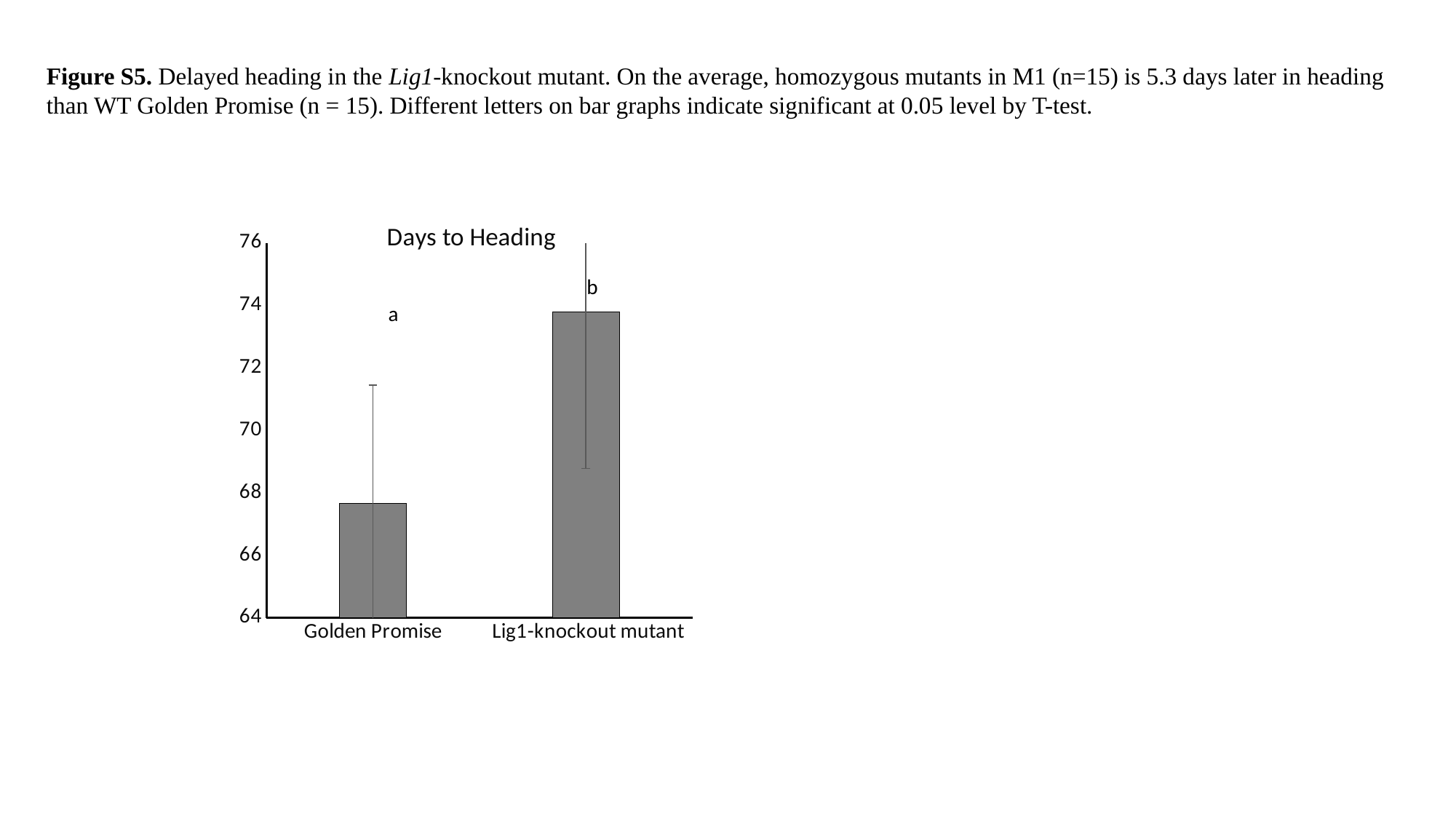

Figure S5. Delayed heading in the Lig1-knockout mutant. On the average, homozygous mutants in M1 (n=15) is 5.3 days later in heading than WT Golden Promise (n = 15). Different letters on bar graphs indicate significant at 0.05 level by T-test.
### Chart: Days to Heading
| Category | |
|---|---|
| Golden Promise | 67.6666666666667 |
| Lig1-knockout mutant | 73.8 |b
a

## Slide 6
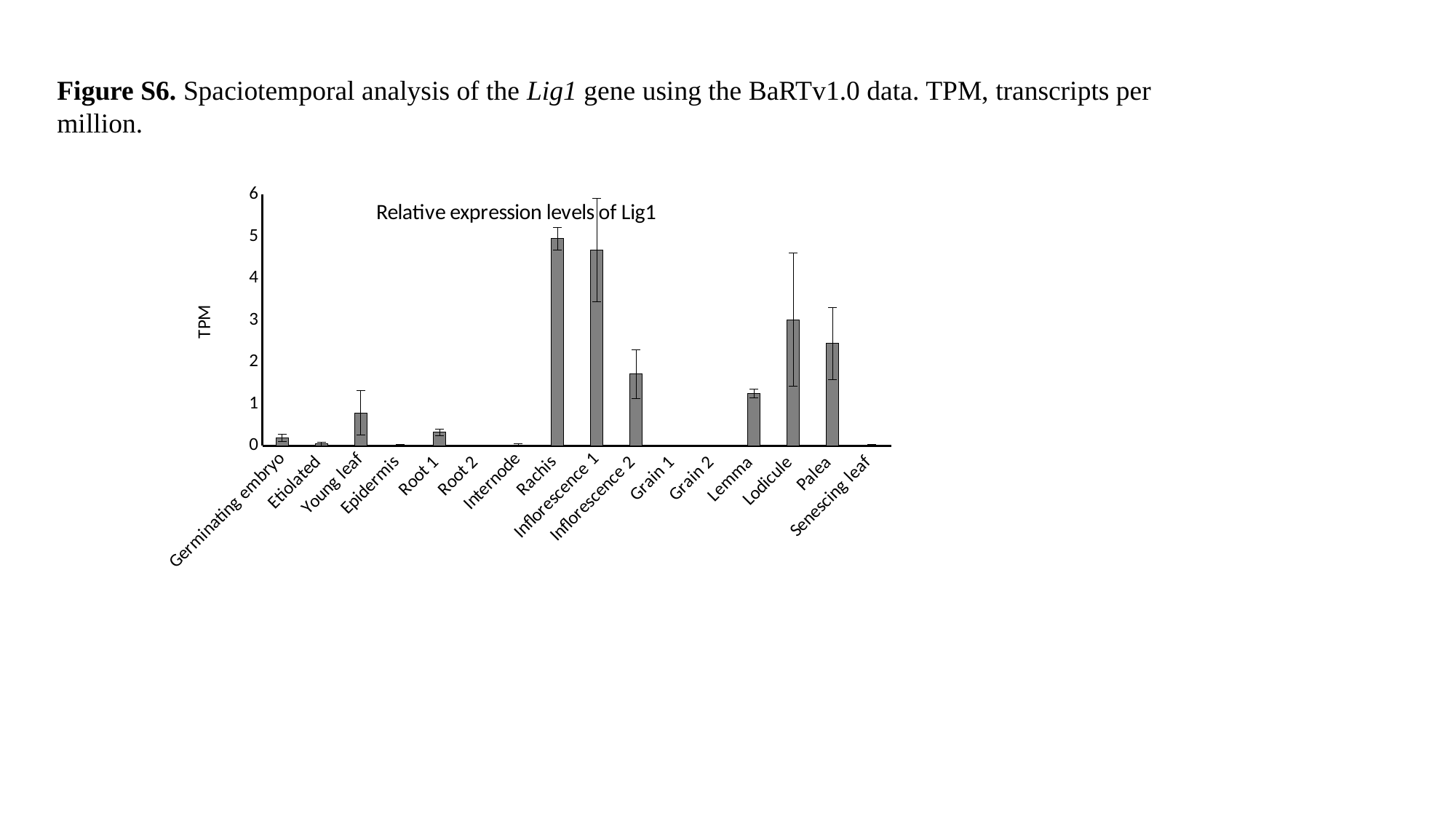

Figure S6. Spaciotemporal analysis of the Lig1 gene using the BaRTv1.0 data. TPM, transcripts per million.
### Chart: Relative expression levels of Lig1
| Category | |
|---|---|
| Germinating embryo | 0.1963113333333333 |
| Etiolated | 0.04390623333333333 |
| Young leaf | 0.7880826666666666 |
| Epidermis | 0.010656866666666667 |
| Root 1 | 0.320644 |
| Root 2 | 0.0 |
| Internode | 0.017257133333333334 |
| Rachis | 4.94646 |
| Inflorescence 1 | 4.681703333333333 |
| Inflorescence 2 | 1.7133366666666667 |
| Grain 1 | 0.0 |
| Grain 2 | 0.0 |
| Lemma | 1.2581733333333334 |
| Lodicule | 3.0137099999999997 |
| Palea | 2.4437533333333334 |
| Senescing leaf | 0.014186333333333334 |
