# Supplementary material for: A SQUAMOSA promoter binding protein‐like transcription factor controls crop ideotype for high productivity in barley
Source: Plant Direct. 2022 Sep 9;6(9):e450. doi: 10.1002/pld3.450 (PMC9477381; doi:10.1002/pld3.450)
Supplement: Supplementary file 2 — Table S1. Marker sequences used for genetic mapping and mutation analysis of the Lig1 gene. Table S2. Predicted genes in the Lig1 region. Table S3. Tissue sampling for Barley Reference Transcript (BaRTv1.0) Dataset (Jayakodi et al., 2020). Table S4. Primer sequences used for qRT‐PCR analysis. [file PLD3-6-e450-s002.pptx]

## Slide 1
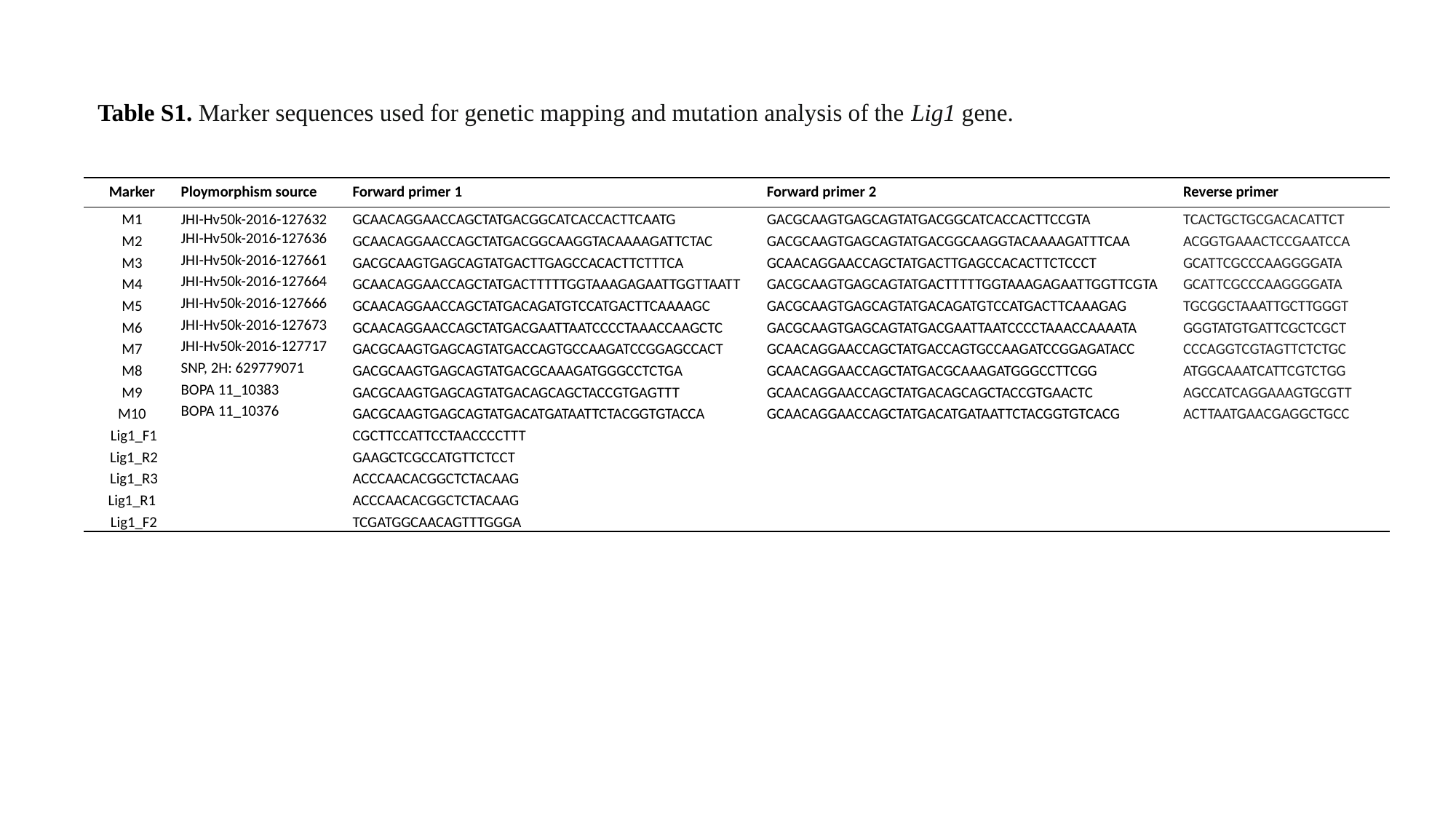

Table S1. Marker sequences used for genetic mapping and mutation analysis of the Lig1 gene.
| Marker | Ploymorphism source | Forward primer 1 | Forward primer 2 | Reverse primer |
| --- | --- | --- | --- | --- |
| M1 | JHI-Hv50k-2016-127632 | GCAACAGGAACCAGCTATGACGGCATCACCACTTCAATG | GACGCAAGTGAGCAGTATGACGGCATCACCACTTCCGTA | TCACTGCTGCGACACATTCT |
| M2 | JHI-Hv50k-2016-127636 | GCAACAGGAACCAGCTATGACGGCAAGGTACAAAAGATTCTAC | GACGCAAGTGAGCAGTATGACGGCAAGGTACAAAAGATTTCAA | ACGGTGAAACTCCGAATCCA |
| M3 | JHI-Hv50k-2016-127661 | GACGCAAGTGAGCAGTATGACTTGAGCCACACTTCTTTCA | GCAACAGGAACCAGCTATGACTTGAGCCACACTTCTCCCT | GCATTCGCCCAAGGGGATA |
| M4 | JHI-Hv50k-2016-127664 | GCAACAGGAACCAGCTATGACTTTTTGGTAAAGAGAATTGGTTAATT | GACGCAAGTGAGCAGTATGACTTTTTGGTAAAGAGAATTGGTTCGTA | GCATTCGCCCAAGGGGATA |
| M5 | JHI-Hv50k-2016-127666 | GCAACAGGAACCAGCTATGACAGATGTCCATGACTTCAAAAGC | GACGCAAGTGAGCAGTATGACAGATGTCCATGACTTCAAAGAG | TGCGGCTAAATTGCTTGGGT |
| M6 | JHI-Hv50k-2016-127673 | GCAACAGGAACCAGCTATGACGAATTAATCCCCTAAACCAAGCTC | GACGCAAGTGAGCAGTATGACGAATTAATCCCCTAAACCAAAATA | GGGTATGTGATTCGCTCGCT |
| M7 | JHI-Hv50k-2016-127717 | GACGCAAGTGAGCAGTATGACCAGTGCCAAGATCCGGAGCCACT | GCAACAGGAACCAGCTATGACCAGTGCCAAGATCCGGAGATACC | CCCAGGTCGTAGTTCTCTGC |
| M8 | SNP, 2H: 629779071 | GACGCAAGTGAGCAGTATGACGCAAAGATGGGCCTCTGA | GCAACAGGAACCAGCTATGACGCAAAGATGGGCCTTCGG | ATGGCAAATCATTCGTCTGG |
| M9 | BOPA 11\_10383 | GACGCAAGTGAGCAGTATGACAGCAGCTACCGTGAGTTT | GCAACAGGAACCAGCTATGACAGCAGCTACCGTGAACTC | AGCCATCAGGAAAGTGCGTT |
| M10 | BOPA 11\_10376 | GACGCAAGTGAGCAGTATGACATGATAATTCTACGGTGTACCA | GCAACAGGAACCAGCTATGACATGATAATTCTACGGTGTCACG | ACTTAATGAACGAGGCTGCC |
| Lig1\_F1 | | CGCTTCCATTCCTAACCCCTTT | | |
| Lig1\_R2 | | GAAGCTCGCCATGTTCTCCT | | |
| Lig1\_R3 | | ACCCAACACGGCTCTACAAG | | |
| Lig1\_R1 | | ACCCAACACGGCTCTACAAG | | |
| Lig1\_F2 | | TCGATGGCAACAGTTTGGGA | | |

## Slide 2
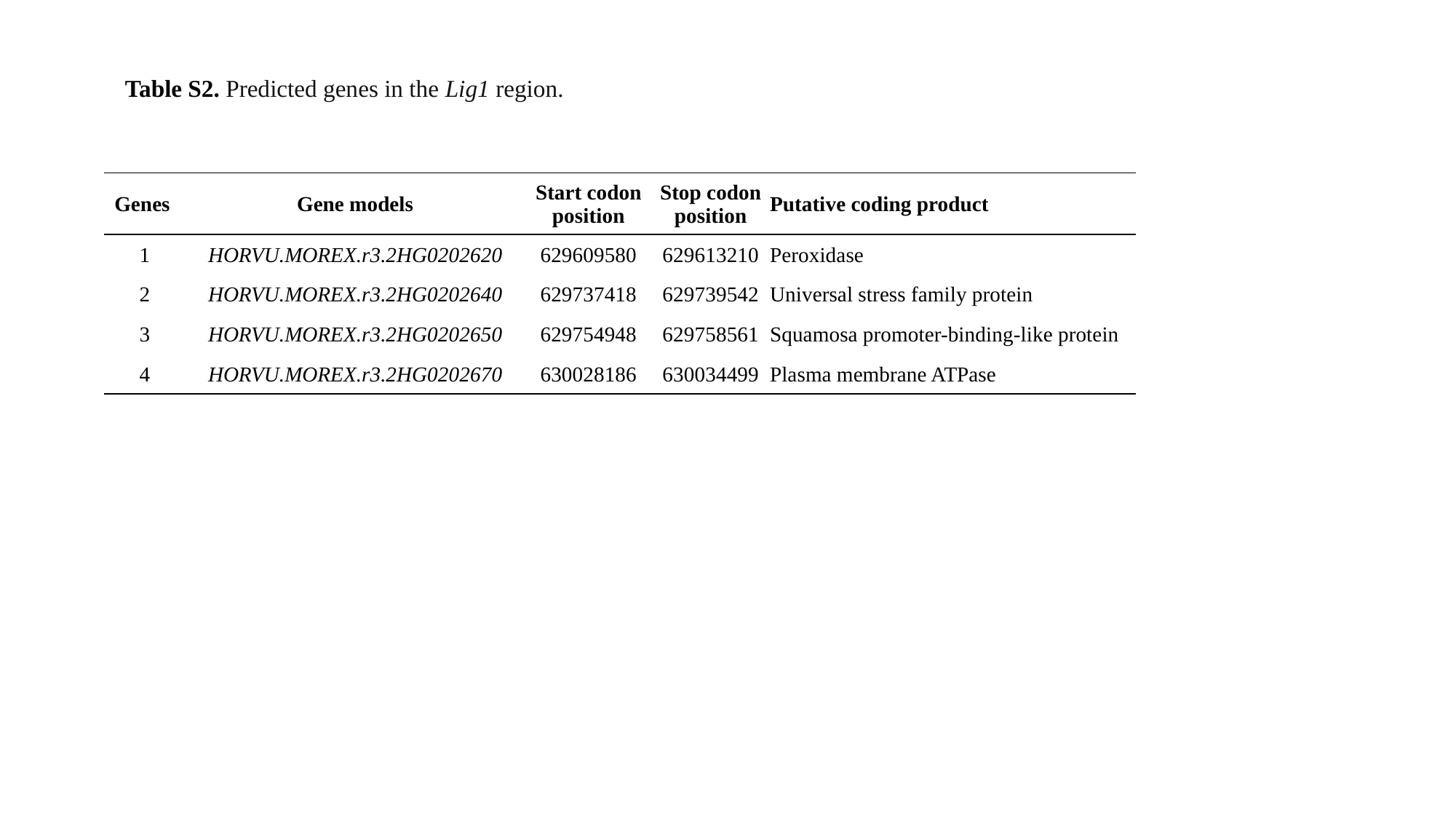

Table S2. Predicted genes in the Lig1 region.
| Genes | Gene models | Start codon position | Stop codon position | Putative coding product |
| --- | --- | --- | --- | --- |
| 1 | HORVU.MOREX.r3.2HG0202620 | 629609580 | 629613210 | Peroxidase |
| 2 | HORVU.MOREX.r3.2HG0202640 | 629737418 | 629739542 | Universal stress family protein |
| 3 | HORVU.MOREX.r3.2HG0202650 | 629754948 | 629758561 | Squamosa promoter-binding-like protein |
| 4 | HORVU.MOREX.r3.2HG0202670 | 630028186 | 630034499 | Plasma membrane ATPase |

## Slide 3
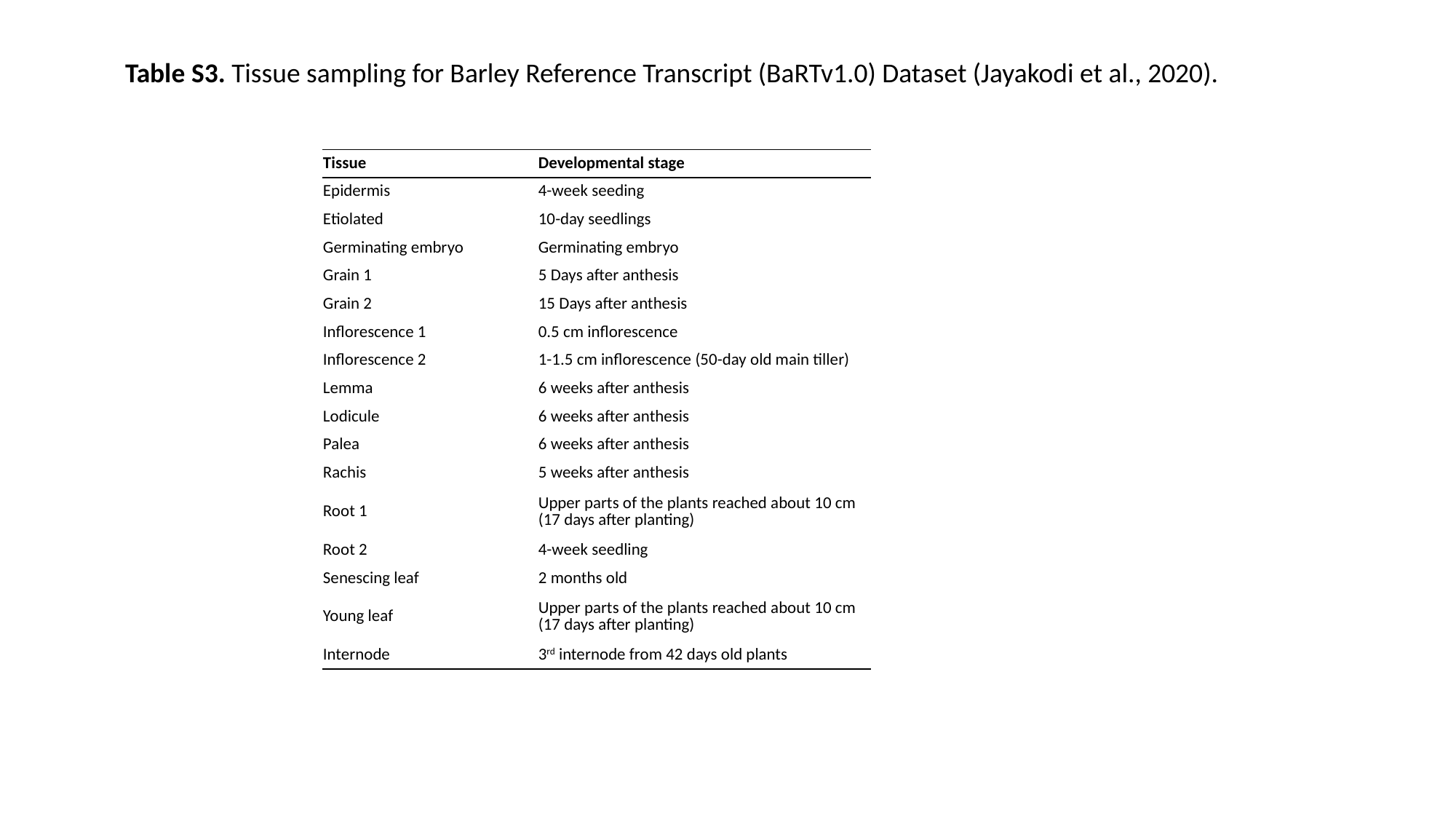

Table S3. Tissue sampling for Barley Reference Transcript (BaRTv1.0) Dataset (Jayakodi et al., 2020).
| Tissue | Developmental stage |
| --- | --- |
| Epidermis | 4-week seeding |
| Etiolated | 10-day seedlings |
| Germinating embryo | Germinating embryo |
| Grain 1 | 5 Days after anthesis |
| Grain 2 | 15 Days after anthesis |
| Inflorescence 1 | 0.5 cm inflorescence |
| Inflorescence 2 | 1-1.5 cm inflorescence (50-day old main tiller) |
| Lemma | 6 weeks after anthesis |
| Lodicule | 6 weeks after anthesis |
| Palea | 6 weeks after anthesis |
| Rachis | 5 weeks after anthesis |
| Root 1 | Upper parts of the plants reached about 10 cm (17 days after planting) |
| Root 2 | 4-week seedling |
| Senescing leaf | 2 months old |
| Young leaf | Upper parts of the plants reached about 10 cm (17 days after planting) |
| Internode | 3rd internode from 42 days old plants |

## Slide 4
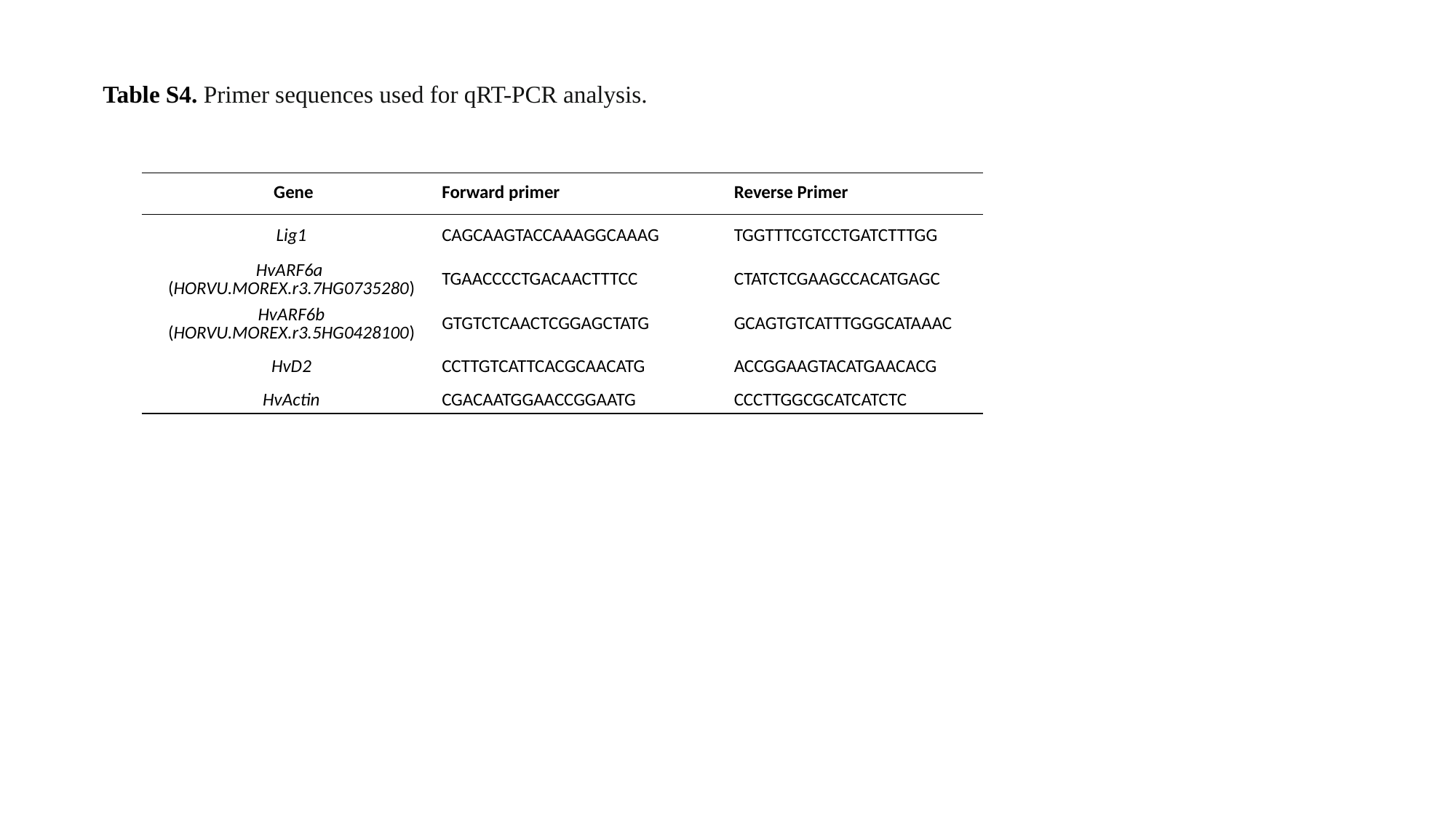

Table S4. Primer sequences used for qRT-PCR analysis.
| Gene | Forward primer | Reverse Primer |
| --- | --- | --- |
| Lig1 | CAGCAAGTACCAAAGGCAAAG | TGGTTTCGTCCTGATCTTTGG |
| HvARF6a (HORVU.MOREX.r3.7HG0735280) | TGAACCCCTGACAACTTTCC | CTATCTCGAAGCCACATGAGC |
| HvARF6b (HORVU.MOREX.r3.5HG0428100) | GTGTCTCAACTCGGAGCTATG | GCAGTGTCATTTGGGCATAAAC |
| HvD2 | CCTTGTCATTCACGCAACATG | ACCGGAAGTACATGAACACG |
| HvActin | CGACAATGGAACCGGAATG | CCCTTGGCGCATCATCTC |
